# Surface Roughness Effects on the Broadband Reflection for Refractory Metals and Polar Dielectrics

**DOI:** 10.3390/ma12193090

**Published:** 2019-09-22

**Authors:** Lina Cao, Kursat Sendur

**Affiliations:** Faculty of Engineering and Natural Science, Sabanci University, 34956 Istanbul, Turkey

**Keywords:** emissivity, surface roughness effects, polar dielectric, broadband reflection, optical properties, extreme environments

## Abstract

Random surface roughness and surface distortions occur inevitably because of various material processing and fabrication techniques. Tailoring and smoothing the surface roughness can be especially challenging for thermomechanically stable materials, including refractory metals, such as tungsten (W), and polar dielectrics, such as silicon carbide (SiC). The spectral reflectivity and emissivity of surfaces are significantly impacted by surface roughness effects. In this paper, we numerically investigated the surface roughness effects on the spectral reflectivity and emissivity of thermomechanically stable materials. Based on our results, we determined that surface roughness effects are strongly impacted by the correlation length of the Gaussian surface. In addition, our results indicate that surface roughness effects are stronger for the materials at the epsilon-near-zero region. Surface roughness effects are stronger between the visible and infrared spectral region for W and around the wavelength of 12 μm for SiC, where plasma frequency and polar resonance frequency are located.

## 1. Introduction

Optical structures are known for their ability to control the spectral reflectivity and emissivity of surfaces [1,2]. With the recent advances in solar thermal [3] and thermophotovoltaic [4] systems and broadband reflection/emission [5] applications, the engineering of surface structures, especially for materials that can sustain high temperature, has become essential. Deterministic surface structures, including 2D and 3D photonic crystals [6] and meta-surfaces [7], have been widely explored to tailor the spectral response of the surfaces to the incident solar spectrum [8,9], increasing the important metrics such as emission efficiency [10].

Random surface roughness and surface distortions occur inevitably as a result of material processing and fabrication techniques [11,12]. For most situations, it is hard to get a completely smooth surface. In some cases, such as the surface roughness of metals, there are reports of improvement of the solar cell energy trapping [11], and the subsequent enhancement in subwavelength imaging [13,14,15]. However, in many cases, the surface roughness impacts the system performance negatively. For example, it was reported that surface roughness decreases the thermal conductivity for Si nanowires [16]. As the impact of surface roughness on the system is crucial, it is important to quantify the impact of surface roughness.

Tailoring and smoothing the surface roughness can be challenging for thermomechanically stable materials, including refractory metals such as tungsten (W) and polar dielectrics, such as silicon carbide (SiC). These materials address problems like corrosion, adhesion, durability, and degraded reflectance performance due to damage, which are some issues that are encountered in various coatings [17,18,19]. Patterned surface structures, such as pyramidal structures [20], blazed gratings [11], and complex square gratings [21] of W, have been investigated by numerical approaches, such as finite difference time domain (FDTD), and experimentally demonstrated to get high absorption efficiencies. Periodic thin-film dielectric coatings deposited over refractory metals were also proposed to reduce absorption [22]. Extending such investigations of refractory metals of deterministic structures to refractory metal surfaces with random surface roughnesses is of interest. As the topology of random surface roughness is quantified by the power spectral density (PSD), the modeling and simulation of random roughness with direct numerical approaches such as FDTD or finite element method (FEM) is challenging.

In the literature, the effect of surface roughness on the interaction of electromagnetic waves with various surfaces has been investigated in detail for various other applications [23,24,25,26]. Both experimental and theoretical studies of the interaction of rough surfaces with electromagnetic waves have led to significant improvements at various spectral regions [27,28,29,30,31,32,33,34]. For example, there is a very large and established literature regarding surface roughness effects over ocean surfaces [35,36,37,38]. Complicated and sophisticated models have been developed in the context of maritime communications, and scattering of electromagnetic waves over rough ocean surfaces, originating due to environmental effects such as wind, have been investigated in detail [29,35,36,37,38,39]. A better understanding of surface roughness effects due to environmental factors has led to a significant improvement of wave propagation for maritime communications. Similarly, the surface roughness of soil surfaces has a profound impact on the signals and signatures of remote sensing applications involving terrestrial applications and buried objects. To understand such effects, the interaction of infrared radiation with rough soil surfaces has been studied extensively [40,41,42,43]. Surface roughness effects in solar cells have attracted significant interest, as surface texturing in solar cells has been shown to improve the efficiency of solar cells [44,45]. In addition to these studies, another area that the interaction of rough surfaces and electromagnetic waves have attracted significant interest is the study surface plasmon excitation with rough surfaces [46,47,48,49,50,51,52].

In terms of experimental studies and applications on rough surface scattering from thin films, there have been investigations on surface roughness effects aluminum and copper surfaces [27]. In addition, various groups have investigates surface roughness effects on plasmonic materials, such as gold and silver [46,47]. Experimental studies related to surface roughness effects in solar cells have attracted significant interest, since surface texturing in solar cells have been shown to improve the efficiency of solar cells [44,45]. In the literature, however, there is a lack of studies on the surface roughness effects of refractory metals.

Despite the established literature on rough surface scattering from surfaces, surface roughness effects on thermomechanically stable materials have been largely ignored. With the increasing interest in thermomechanically stable materials, including refractory metals and polar dielectrics, an investigation of surface roughness effect on their broadband reflection spectrum is necessary. To address these issues, in this study, we developed a model and investigated the impact of surface roughness on the spectral reflectivity under a broadband illumination. In this paper, we applied small perturbation method/small slope approximation (SPM/SSA) methods [35] to study reflectivity change in a broadband spectrum caused by Gaussian roughness for thermomechanically stable materials. The effects from the optical properties of materials and the surface roughness structures are studied and discussed. Special attention is paid to polar SiC and W.

## 2. Computational Methodology

**Figure 1:**
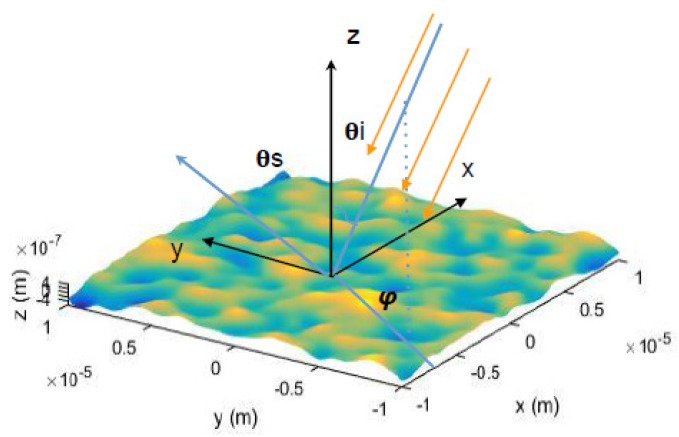
Schematic illustration of the surface roughness and the light scattering system.

To understand the random surface roughness effect, a rough surface configuration shown in Figure 1 is considered. Light illuminates the surface from vacuum with an incident angle, $\theta_{i}$, and azimuth angle, $\phi_{i}$. As the surface is not flat, the use of different facet angles would cause light to deflect in different directions. The directions of scattered light are thus labeled with $\theta_{s},$ $\phi_{s}$. To study the broadband reflectivity and emissivity, we considered normal incidence of illumination, where $\theta_{i}=0$. Surface roughness is quantified in terms of the root-mean-square (RMS) of surface height σ and the transverse correlation length *l*. The power spectral density (PSD) of the surface is mathematically the Fourier transformation of the surface height self-correlation function [53]. The integration of PSD function over k-space results in the room-mean-square (RMS) of surface height, thus σ. Sinusoidal surface shapes will show as a delta function for the PSD. For a Gaussian surface, the roughness is randomly distributed with a PSD function, which follows a Gaussian distribution. In this study, we consider a Gaussian surface, the PSD of which is given as [53]

(1) $$W\left(k_{x},k_{y}\right)=\frac{l^{2}\sigma^{2}}{4\pi }e^{-\frac{\left(k_{x}^{2}+k_{y}^{2}\right)l^{2}}{4}}=\frac{l^{2}\sigma^{2}}{4\pi }e^{-\frac{k_{\rho }^{2}l^{2}}{4}}$$

where *l* is transverse correlation length, $\sigma^{2}$ is the surface height variance, and $k_{\rho }^{2}=k_{x}^{2}+k_{y}^{2}$ is the wave-vector in the radial direction. In Equation (1), we assumed the same correlation length along the x- and y-directions, which is $l_{x}=l_{y}$, thus the random roughness is isotropic with no azimuthal angle dependence.

Theoretical methods in studying the surface roughness effect includes Lippmann–Schwinger equation [54], small perturbation method [23], and small slope approximation (SSA) [35]. The numerical methods used herein include moments of momentum (MoM) and Monte Carlo [55]. Among these techniques, the SPM/SSA method, up to second order, has been proven to be valid for studying the surface roughness with small variance or small slopes. It has been widely used in the remote sensing of thermal temperature of sea surfaces [35]. In the literature, numerical techniques have been used for studying scattering from rough surfaces, including finite difference time domain (FDTD) [20], finite element method (FEM) [56], method of moments and T-Matrix methods [13,14,15], and Monte Carlo approaches [57]. The SPM/SSA technique used in this manuscript offers advantages for the problems that we address. Numerical approaches, such as FDTD- or FEM-based methods, provide solutions for deterministically described rough surfaces, whereas the SPM/SSA technique provides solutions for stocastically described rough surfaces. Compared to SPM/SSA, typical MoM or Monte Carlo approaches are more time-consuming. The SPM/SSA method, up to the second order, contains the scattered beam contribution from both coherent and incoherent terms, thus resumes energy conservation [58]. In this paper, we followed the formulation from the work by the authors of [29,35].

In this study, we present a theoretical investigation on rough surface scattering A simplified description of the method is discussed here. The reflection from the rough surface is given as [35]

(2) $$R=\left(\begin{array}{c} |R_{hh}^{0}{|}^{2}\\ |R_{hv}^{0}{|}^{2}\\ 0\\ 0 \end{array}\right)+\int_{0}^{\infty }d\beta \int_{0}^{2\pi }d\varphi^{\prime }C\left(k_{0}\beta ,\varphi^{\prime }\right)\left(\begin{array}{c} g_{h}\left(\beta ,\theta_{i},\varphi_{i},\epsilon ,\varphi \right)\\ g_{v}\left(\beta ,\theta_{i},\varphi_{i},\epsilon ,\varphi \right)\\ g_{U}\left(\beta ,\theta_{i},\varphi_{i},\epsilon ,\varphi \right)\\ g_{V}\left(\beta ,\theta_{i},\varphi_{i},\epsilon ,\varphi \right) \end{array}\right)$$

where $|R_{hh}^{0}{|}^{2}$ and $|R_{hv}^{0}{|}^{2}$ corresponds to the Fresnel reflection coefficients for horizontally and vertically polarized light incidence, respectively. In Equation (2), the contributions from the $|R_{hh}^{0}{|}^{2}$ and $|R_{hv}^{0}{|}^{2}$ terms account for the reflectivity of a flat surface. Reflectivity changes due to the surface roughness are accounted in the second term in Equation (2), where $\beta =k_{\rho }/k_{0}$ and [35]

(3) $$C\left(k_{\rho },\varphi \right)=k_{\rho }^{4}W\left(k_{\rho },\varphi \right)$$

By substituting Equation (3) into Equation (1), we obtain

(4) $$C\left(k_{\rho },\varphi \right)=\frac{k_{\rho }^{4}l^{2}\sigma^{2}}{4\pi }e^{-\frac{k_{\rho }^{2}l^{2}}{4}}$$

As weighting functions, *g*, are functions of $\varphi_{i}-\varphi$ alone, integral over φ makes the dependent on $\varphi_{i}$ to be vanished. In our calculations, we choose $\varphi_{i}=0$. The *g* function in Equation (2) can be expanded into its Fourier series as

(5) $$g_{\gamma ,n}\left(\theta_{i},\epsilon ,\beta \right)=\frac{1}{2\pi }\int_{0}^{2\pi }d\varphi^{\prime }e^{in\varphi }g_{\gamma }^{\prime }\left(\theta_{i},\epsilon ,\beta ,\varphi^{\prime }\right)$$

Meanwhile, function C, given in Equation (4), can be expanded into its Fourier series as

(6) $$C_{n}\left(k\beta \right)=\int_{0}^{2\pi }d\varphi^{\prime }e^{-in\varphi^{\prime }}C\left(k\beta ,\varphi^{\prime }\right)$$

Therefore, the roughness caused reflection change can be written as

(7) $$\Delta R=\int_{0}^{\infty }d\beta g_{\gamma ,0}^{\prime }\left(\beta \right)C_{0}\left(k_{0}\beta \right)+2\sum_{n=1}^{\infty }\int_{0}^{\infty }d\beta Re\left\{g_{\gamma ,n}^{\prime }\right\}C_{n}\left(k_{0}\beta \right)$$

Considering the large range of wavevectors that are going to be analyzed, each azimuth harmonic term in Equation (7) is rewritten to be an linear integration over $\log_{10}\beta$, which is

(8) ∫0∞dβRe{gγ,n′(β)}Cn(k0β)=(ln10)∫-∞∞d(log10β)βRe{gγ,n′(β)}Cn(k0β)

In the following sections, the surface roughness-caused reflection change ΔR is calculated using Equation (7) and Equation (8). In Equation (7), ΔR is decomposed into two terms, $g_{\gamma ,n}^{\prime }\left(\beta \right)$ and $C_{n}\left(k_{0}\beta \right)$, given by Equation (5) and Equation (6), respectively. Here, the weighing function, *g*, is determined by the material optical properties, and *C* depends only on the surface profiles. The magnitude of *g* values are derived from scattering coefficients; the full description is illustrated in the work by the authors of [29].

## 3. Results and Discussions

As the PSD of sinusoidal surfaces are delta functions at the corresponding wave-vector *k* [53], each value of the weighting function *g* at a given *k* determines the reflection change caused by the corresponding sinusoidal surface. For the Gaussian surface in this paper, we take the 0th harmonic of the weighting function into account. As a result, weighting functions depend only on the optical permittivity of materials for a chosen beam incident angle. To illustrate the different scattering properties of different materials in this study, the weighting functions for materials with artificial dielectric constants are presented. Refractive index of material is represented as n+ik, where *n* and *k* correspond to the real and imaginary parts of the dielectric constant, respectively.

**Figure 2:**
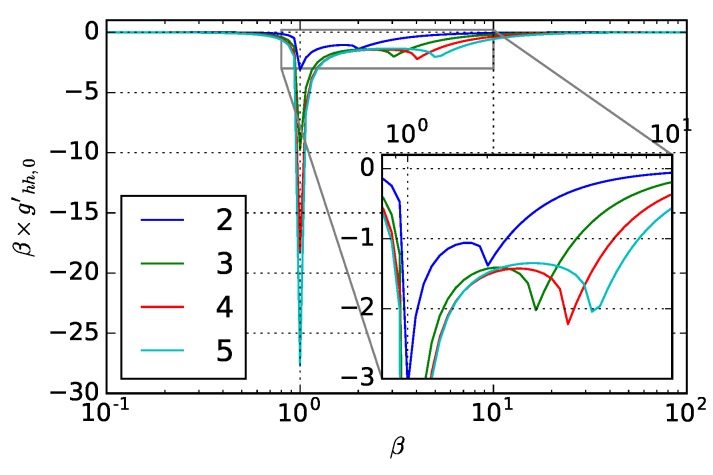
0th-order weighting function magnitude with respect to optical constant for materials with k=0. The legend in the figure shows the corresponding *n* value for each curve. Inset plot corresponds to the region in the gray box.

Figure 2 shows the normal incidence weighting functions for dielectrics with k=0, where the materials are transparent with no absorption. As can be seen, there is a strong resonance peak at β=1; a secondary resonance peak happens around the β=n. At β=1, the corresponding space periodicity of the surface has the same periodicity of incident wavelength λ, where perfect phasing matching is provided for the reflected waved, therefore, causing less reflection. For the secondary resonance peak, the corresponding periodicity is λ/n, which is the wavelength of beam inside the material. As there is no absorption from the material, reflection changes are purely brought by the roughness periodicity-caused momentum change. When the surface roughness gets larger than the incident wavelength, it behaves like smooth surface and weighting function magnitude gets close to 0. Similarly, when the surface roughness gets very small, the weighting function also decreases. Surface roughness with the periodicity between incident wavelength and the wavelength in the material have strong effect of the surface reflection and the effect slowly decreases as roughness gets smaller.

**Figure 3:**
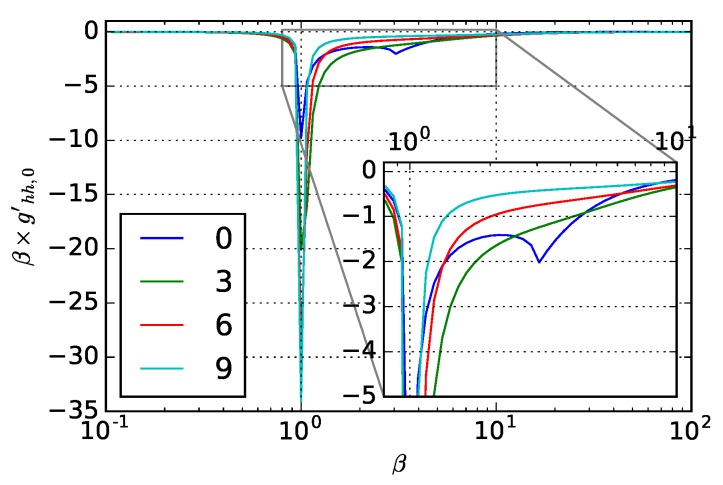
0th-order weighting function magnitude with respect to dielectric constant for materials with $n_{r}=3$. The legend in the figure shows the corresponding *k* value for each curve. Inset plot corresponds to the boxed region.

The materials experience absorption of the penetrated beam, which can be quantified by the imaginary part of permittivity, $\epsilon =n^{2}=n^{2}-k^{2}+2nki$, at n=k,ϵ=0. For metals, this is caused by the electron oscillations and the corresponding frequency is called plasma frequency $\omega_{p}$. For polar dielectrics, phonon resonances can also cause an epsilon-near-zero region, which corresponds to the so-called Reststrahlen band [59]. Here, artificial material with fixed $n_{r}=3$ is chosen and the corresponding weighting functions for varying *k* values are calculated and plotted in Figure 3. The blue curve corresponds to k=0, where two resonance peaks at the locations of λ and λ/n exist in the curve as discussed above. As *k* increases, beams that have penetrated the material experience energy absorption, providing extra phase and momentum change of optical beams; therefore, the second resonance peak which corresponds to the light wavelength inside the material decreases and disappears. Due to the absorption and reemission, the reflected beams experience more complicated phase change compared to pure dielectrics with k=0. As *k* increases and becomes larger than $n_{r}$, ϵ becomes negative. Surface behaves like perfect electrical conductor with high reflectivity, the influence from surface roughness under this condition is also relatively small. Weighting function shows as one sharp peak at the incident λ.

Considering function C(β), the maximum magnitude happens at $\frac{\partial C}{\partial \beta }=0$, where $\frac{2\sqrt[{}]{2}}{k_{0}l}=\frac{\sqrt[{}]{2}}{\pi }\frac{\lambda_{0}}{l}$ and the maximum value is defined as

(9) $$C_{max}=\frac{16}{\pi e^{2}}{\left(\frac{\sigma }{l}\right)}^{2}$$

**Figure 4:**
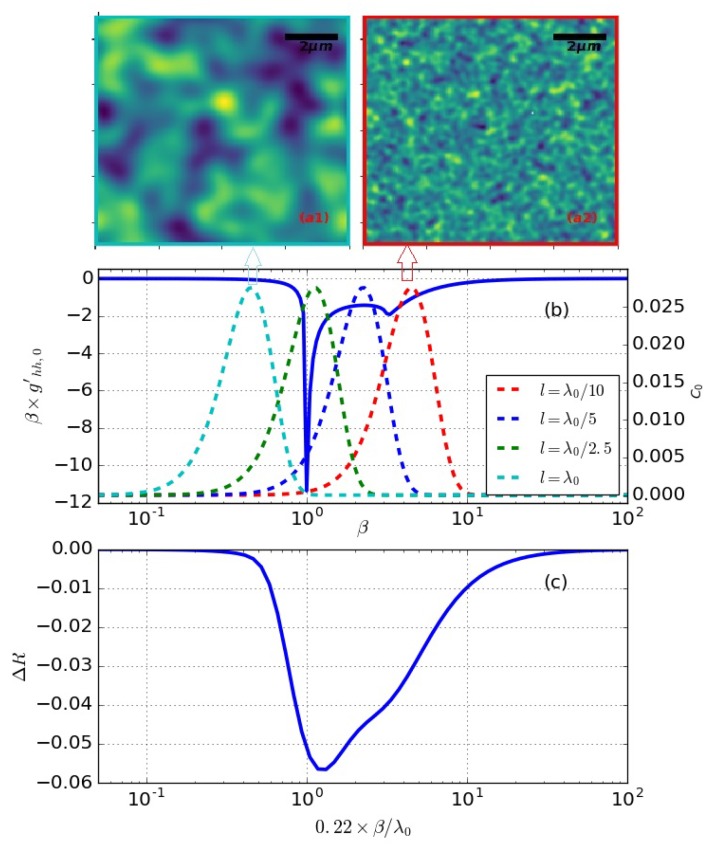
Illustration of $C_{0}$ functions for Gaussian surfaces overlapped with the weighting function and corresponding surface profile. The top panels (**a1**,**a2**) show two example surface height profiles. Panel (**b**) shows weighting function with four $C_{0}$ functions. Panel (**c**) shows roughness caused surface reflection change ΔR versus the 0.22β/l, which is the location of $C_{0}$ function maximum.

Thus, the maximum value of C for Gaussian surfaces is proportional to ${\left(\frac{\sigma }{l}\right)}^{2}$. In Figure 4, we plotted four *C* functions with the transverse correlation lengths chosen to be λ/10,λ/5,λ/2.5,λ, respectively. For all these curves, σ=l/5. σ/l are kept to be the same thus the *C* curves have the same maximum values according to Equation (9). σ/l shows the general slope of the surface roughness, for SPM/SSA method to be valid, σ/l of smaller than 4 is necessary [58]. As illustrated in Figure 4, the peak positions of function *C* moves to the smaller length scales section as *l* gets smaller. At the location β=1, which is the resonance position for weighting functions, the maximum *C* happens at $l/\lambda_{0}=\pi /\sqrt[{}]{2}\approx 2.22$. Due to the fast fall off of the weighting functions, when $l=\lambda_{0}$ (cyan curve in Figure 4), panel (b) overlaps between the function *C*, and the weighing function becomes too small, thus the reflection change caused by the roughness can be neglected. In other words, the roughness length scale is too big for the incident light to detect and the reflection value is close to smooth flat surface. When *l* is less than $\lambda_{0}$, the weighting functions do not fall as fast, and the maximum overlap between these two function happens when l is between λ/n and λ/2.22. Panel (c) in Figure 4 plotted the reflection change for Gaussian surfaces with different correlation lengths. The x-axis is the correlation length *l* in the unit of λ/2.22, in other words, the location of $C_{max}$ for surface curves. As illustrated, the maximum ΔR happens with the maximum overlap between the surface *C* function and the weighting function, which is in between λ/n and λ/2.22. Two example surface height profiles are shown in Figure 4, panel (a). At the left profile, panel a(1), l=λ/2.5=600 nm, and for the right profile l=λ/10=150 nm. The *C* function for these two surface profiles are the dotted green and dotted red curve respectively. As stated previously, σ/l are kept the same for these illustrations, therefore, smaller *l* corresponds to smaller defects.

After a brief discussion of the weighting function and the correlation functions, we discuss the surface roughness effects for refractory metals and polar dielectrics, which are popular materials for high temperature applications. Here, we chose tungsten and SiC as two examples, and analyzed the surface reflection change caused by random surface roughness.

**Figure 5:**
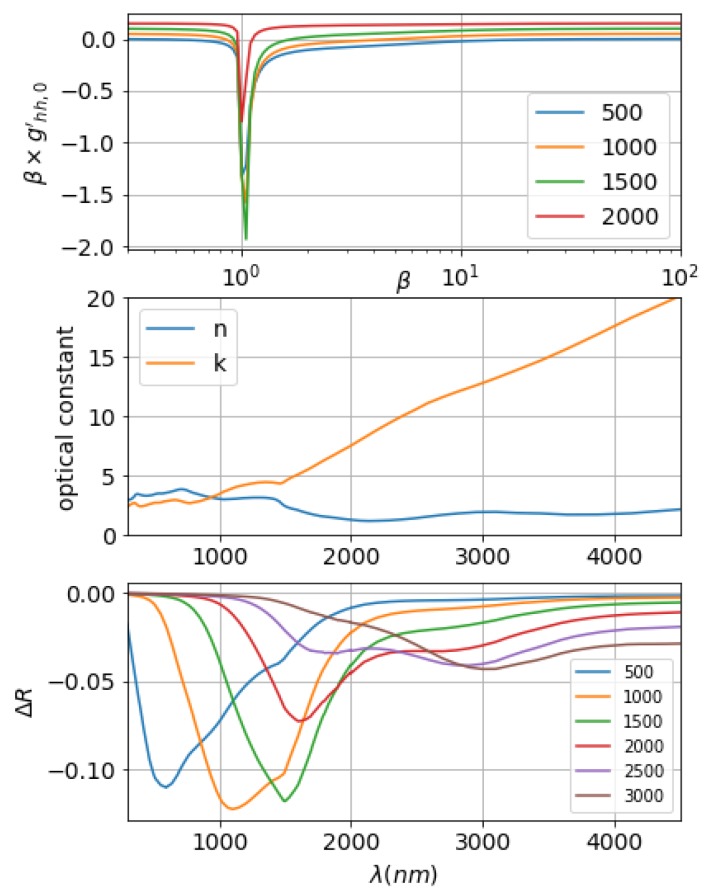
Random roughness effects on tungsten surface. Top panel: weighting function for wavelengths λ = 500 nm, 1000 nm, 1500 nm, and 2000 nm. Middle panel: optical dielectric constants for W at the visible and near-infrared range. Bottom panel: Reflection spectrum change of W surface for Gaussian surface with different correlation lengths. Legend: 2.22l in nm.

Similar to other refractory metals, W shows a high reflection at far-infrared range, whereas it experiences a considerable absorption in the visible and near-infrared [22]. We pick four wavelengths: 500 nm, 1000 nm, 1500 nm, and 2000 nm. We calculated the corresponding weighting functions, which are plotted in the top panel of Figure 5. With reference to the middle panel of Figure 5, at λ=500 nm, n>k, ϵ>0; at λ=1000 nm, *n* gets smaller compared to 500 nm, *k* is bigger, and ϵ gets close to 0. As can be seen from the second panel, the weighting function for λ = 500 nm, 1000 nm is very close in magnitude. At λ=1500 nm, the magnitude of the weighting function starts to get smaller especially when β gets away from the incident wavelength resonance location. At λ=2000 nm, ϵ gets far negative, where weighting function gets a lot smaller in magnitude compared to the previous three wavelengths. As the maximum reflection change point is closely correlated with the incident λ, if we look at the problem from the broadband spectrum point, for a fixed topology of surface roughness, its influence on the spectrum will mainly be around the l/2.22 location. Keeping this idea in mind, the broadband spectrum for W Gaussian surfaces are calculated and plotted in the bottom panel. The legend in the panel shows the maximum location of the chosen *C* function, in other words, l/2.22. For the first three curves—*l*/2.22 = 500 nm, 1000 nm, and 1500 nm—the maximum ΔR value location is around the corresponding wavelengths. For all of these curves, σ/l are kept the same for each surface profile, therefore σ will be larger for bigger *l* curves. It shows in the curve that the ΔR values smooth out at longer wavelengths. This confirms that when the surface roughness size is very small compared to λ, ΔR depends only on the value of σ [60]. For the red curve, where $C_{max}$ location at 2000 nm, it is expected that one resonance peak would show around λ=2000 nm, however, the resonance peak actually appears at around λ=1500 nm, which is a big resonance shift. Referring to the first three curves, we also see shoulder peaks at around λ=1500 nm. Refer to the top panel in this figure, for Tungsten, epsilon becomes negative at around λ>1000 nm, where surface plasmon resonances (SPR) can be excited. The localized surface plasmon resonance will increase the absorption of the material thus shows as a higher reflection reduction. Gaussian surfaces with one certain transverse correlation length can be decomposed into sinusoidal surfaces with the corresponding periodicity. As the corresponded periodicities for Gaussian surface covers one certain range centered around *l*, shown as the *C* function in Figure 4, if *l* is around $\omega_{p}$, LSPR gets excited and increases absorption. At $C_{max}$ location at 2000 nm, the weighting function for λ=2000 nm is relatively small due to the high negative number of ϵ, the resonance peak matching the periodicity does not get as high as the LSPR peak, thus showing as a resonance shift on the reflection spectrum. As *l* gets larger and further away from the $\omega_{p}$ frequency, corresponded LSPR resonance also gets lower due to the magnitude of *C* function at this resonance frequency gets lower. The LSPR resonance peak can still be seen at $C_{max}=2500$ nm, but disappears at $C_{max}=3000$ nm. At these larger *l*s, the primary resonance peak is also not as sharp and it falls very fast to one smooth value.

**Figure 6:**
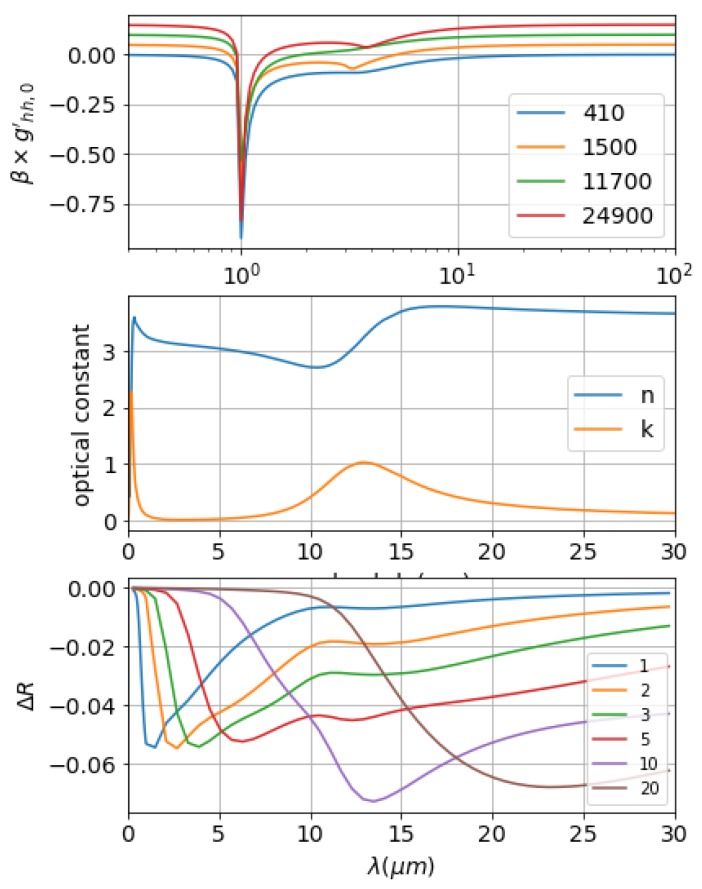
Random roughness effects on SiC surface. Top panel: weighting function for wavelengths λ = 410 nm, 1500 nm, 11,700 nm, 24,900 nm. Middle panel: optical dielectric constants for SiC at the visible and near-infrared range. Bottom panel: Reflection spectrum change of SiC surface for Gaussian surface with different correlation lengths. Legend: 2.22l in μm.

A similar analysis is performed for SiC. Figure 6 illustrates the optical constant, the weighting functions of the selected wavelengths, as well as the broadband reflection change for fixed Gaussian surface topologies. SiC is a lossy dielectric in infrared range. Incident beams penetrates dielectrics thus resulting low reflection. Four wavelengths—λ=410 nm, 1500 nm, 11,700 nm, and 24,900 nm—were chosen to illustrate the weighting functions, as shown in the top panel of Figure 6. Compared to W, the magnitude of weighing functions are smaller. As the magnitude of weighting function of W approaches −20, the magnitude for SiC is −1. Even the *n* values for W and SiC are comparable, as there is significant difference in *k* values. Lower *k* values for SiC means lower absorption for penetrated beams propagating inside the material. As seen in the top panel, the secondary resonance peak shows for the chosen wavelengths. As the differences among the weighting functions are relatively small, roughness caused reflection change are also highly depended on the ration of l/λ, with σ/l fixed. The results are shown in panel 3. As expected, at the long wavelength range, the surface reflection change increases as σ increases. The maximum reflection change occurs when the maximum overlap of the Gaussian surface C function and weighting function, which is when the $C_{max}$ locates at λ/2.22. Further, a secondary resonance peak is located around the location of λ between 10,000 nm and 15,000 nm, where the lattice oscillations cause extra absorption. These lattice polar resonances behave like Lorentz oscillators for polar dielectric crystals, and the dispersion relation are similar to those of metals [59]. Referring to middle panel, which illustrates the optical constant of SiC, we can clearly see one resonance peak at λ≈ 10 μm–15 μm. For some polar dielectrics, such as 4H-SiC, surface phonon resonance (SPhR) modes can be excited during the Reststrahlen band, where results epsilon near to zero and negative Re(ϵ). According to our previous discussion, the surface roughness for these materials will be expected to experience some higher reflection change at the resonant frequency location.

After this detailed discussion on various materials, the main findings and differences of each case can be summarized as follows. For the lossless case, the weighting function has two resonance peaks, corresponding to the incident wavelength and wavelength of beam inside the material, however the magnitude of weighting functions are limited compared to lossy materials. For the lossy materials that we studied, a comparison of W and SiC suggests that SiC is more dielectric as *n* > *k* for interested spectrum range for SiC (400 nm to 2 um); thus, for the same σ, which is height variance, roughness causes bigger reflection change for W, especially at the near-infrared range, where epsilon of W is close to 0. For SiC, the roughness caused by the spectrum change can show the magnification in the 10 um to 15um range, which corresponds to the phonon resonance in SiC; however, as SiC is still dielectric at this range, the magnification is limited.

## 4. Conclusions

In this paper, we applied the SPM/SSA method to study the random surface roughness effects on the reflectivity of refractory metals and polar dielectrics. This method enables the decomposition of surface roughness topography and material properties. Larger values of *n* show a higher roughness effect as well as a broader length scale range, which are illustrated as weighting functions. Transparent dielectrics with low *k* values experience two resonance locations on the weighting function, corresponding to the periodicity of roughness same as incident wavelength and the wavelength in material. The absorption increase (*k* increase) for materials will increase the weighting function in magnitude, but the secondary resonance peak weakens and disappears. The maximum roughness effect happens when *k* is similar, in terms of magnitude, to *n*, where epsilon is close to zero.

For refractory metal W, as *n* and *k* are relatively close in terms of magnitude at the visible to infrared range, surface roughnesses with the corresponding correlation lengths cause higher absorption change with same roughness height variance. At the far-infrared region, where flat W surface experience high reflectivity, the reflection change due to surface roughness is also limited. For SiC, as the magnitude *k* is smaller than *n* at infrared, surface roughness caused reflection change is limited; however, polar resonance at λ around 12 μm causes one resonance peak at this location for the surface roughness effect. Our results indicate that maximum impact on the reflection from a rough material surface is observed when the correlation length of the random surface roughness satisfies $l=2.22\lambda_{0}$ condition and the operation frequency matches the internal absorption frequencies (plasma frequency and phonon frequency).
